# Reforming the innovation system to deliver affordable medicines: a conceptual framework of pharmaceutical innovation as a complex adaptive system (forest) and theory of change

**DOI:** 10.1080/20523211.2024.2436899

**Published:** 2025-01-16

**Authors:** Suerie Moon, Adrian Alonso Ruiz, Marcela C. F. Vieira, Kaitlin E. Large, Iulia Slovenski

**Affiliations:** a Department of International Relations and Political Science, Graduate Institute of International and Development Studies, Geneva, Switzerland; b Global Health Centre, Graduate Institute of International and Development Studies, Geneva, Switzerland

**Keywords:** Pharmaceutical innovation system, medicines, research and development (R&D), fair pricing, affordability, alternative innovation model, business model, complex adaptive system, pandemic preparedness and response, neglected diseases, rare diseases, antibiotics

## Abstract

**Background:**

The current mainstream pharmaceutical innovation system (PIS) is driven by the market-based logic of charging the highest prices societies will bear. Outcomes include unaffordable medicines, restricted access and pressure on health budgets. How can the innovation system change to deliver fairly-priced medicines?

**Methods:**

We inductively developed a novel conceptual framework of the PIS as a complex adaptive system (CAS) analogous to a forest. We constructed a database of 140 pharmaceutical innovation initiatives that sought to address global public interest objectives such as fair pricing or missing innovation. We found a critical mass of initiatives clustered around four areas: pandemic preparedness, neglected diseases, rare diseases and antibiotics, which we conceptualised as *niches* within the ecosystem. We reviewed the literature on how each niche had emerged and evolved, conducted interviews, and organised workshops with experts on each niche. Finally, we identified from the literature an initial list of ‘levers’ of change in the PIS, supplemented them with additional levers found in each niche, then compared across niches.

**Results:**

We found that actors created niches in the broader system by purposefully problematising an issue, then pulling on one or more of three levers: mobilising new resources, changing the roles of or creating new actors, and/or changing societal norms or legal rules. A wide range of actors – including governments, funders, R&D practitioners, or civil society groups – could pull these levers, and the order in which they were pulled was not fixed, consistent with a CAS.

**Conclusions:**

Parts of the vast pharmaceutical innovation system have changed to deliver more affordable medicines by design. Such change has occurred largely within specialised niches, responding to evolving societal norms about the purpose of pharmaceutical innovation. Actors can achieve larger-scale change by further expanding and/or solidifying these niches through changes to resources, actor roles, norms and rules.

## 1. Background

### 1.1. Introduction

Vaccines, drugs and other health technologies such as diagnostics are critical for protecting human life, health and well-being. As Covid-19 vaccines demonstrated, they can also be critical for economic and national security. Ensuring the development of new technologies for unmet health needs while also ensuring fair pricing and equitable access is a daunting challenge facing all countries.

The global pharmaceutical innovation system (PIS) is vast, complex and flawed. The global market for medicines is projected to reach USD 2.2 trillion by 2028 (IQVIA Institute, 2024), and comprised one-sixth of worldwide health spending in 2020.^1^ This system has yielded major technological and therapeutic advances, such as the ability to cure previously-chronic diseases such as hepatitis C virus (HCV) or to prevent a child from progressively going blind from a genetically-inherited disorder (US Food and Drug Administration, 2017). But these advances have raised serious concerns about the fairness of pricing, with the high initial prices of HCV drugs leading to rationing in even the wealthiest countries (Canary et al., 2015; Iyengar et al., 2016) and prices for gene therapies ranging from $375,000 to over $2 million per patient (Sabatini et al., 2022). In assessing fairness, we rely on Moon’s conceptualisation of a fair price of a medicine as ‘affordable to the buyer while covering the seller’s costs and providing a reasonable profit margin’ (2020).

The current PIS is designed to deliver high prices. While public funding plays a critical role by paying for and de-risking basic and early-stage research (Cleary et al., 2020), private investment largely pays for the later-stage R&D required to bring a product to patients (Røttingen et al., 2013). Private investors require the prospect of sufficiently-attractive profits, which are often driven by high prices that strain the sustainability of health budgets and restrict access to important new medicines worldwide (Morgan et al., 2020). This PIS also fails to meet society’s health needs in other important ways. For example, because it relies heavily on profit incentives, the system does not produce adequate innovation in areas where such incentives are too small or risky, such as for diseases predominantly affecting the world’s poorest people (Pedrique et al., 2013). Second, competition for market share impedes knowledge-sharing, which can unnecessarily retard scientific progress and result in wasteful, redundant research (Mazzoleni & Nelson, 1998). Third, the same monopoly intellectual property (IP) rights that can incent R&D investments can also limit manufacturing and price competition, restricting access to medicines (CIPIH/WHO, 2006). Patent monopolies can also restrict further innovation that would build on the protected knowledge (Jaffe & Lerner, 2004). As a result, scientific progress is skewed towards the health needs of the world’s wealthy, and access to the fruits of such progress is often highly unequal (Swaminathan et al., 2022).

Scholars have argued that new innovation models are needed to address these shortcomings (Moon et al., 2022; Suleman et al., 2020). We found the emergence of such alternative innovation models (AIMs) clustered in four sub-systems – or ‘niches’ – within the broader PIS. Such niches are characterised by norms and rules, resources and/or actors that differ from the mainstream system. However, the emergence and potential of AIMs and niches to deliver affordable medicines prices is underexplored in the literature. This paper seeks to fill this gap by offering a novel conceptual framework of the PIS as a complex adaptive system analogous to a forest, describing the evolution of pharmaceutical innovation niches within the broader ecosystem, analysing brief histories of four such niches, and developing a theory for what drives their creation and evolution. In doing so, we hope to deepen understanding of how actors can change the PIS to deliver fair pricing as a systematic outcome.

### 1.2. Literature review

Our inquiry draws from and builds on the literature on drivers of change in the mainstream PIS and alternative niches within that system.

The literature has frequently conceptualised the pharmaceutical sector as an innovation system, whether based on a geographical or sectoral logic (Freeman, 2002; Nelson, 1993). A central concern has been how to strengthen such systems to achieve national policy goals such as economic development, competitiveness or technological advancement (Chataway et al., 2007; Chung, 2013; Mytelka, 2006; Ni et al., 2017). Alternatively, pharmaceuticals have also usefully been conceived as a sectoral innovation system, one that has become increasingly transnational with the expansion of firms into multiple markets, and the globalisation of IP and regulatory rules (Dutfield, 2020; Malerba, 2002; McKelvey & Orsenigo, 2001).

We follow McKelvey and Orsenigo’s (2001) conceptualizion of pharmaceuticals as a sectoral innovation system, with a heterogeneous mix of actors (e.g. firms, academia, government research funders, purchasers) that has evolved continuously and considerably over its history. The sector has its roots in the nineteenth century textile dye industries of Germany and Switzerland. With the importance of penicillin and anti-malaria drugs to sustain fighting capacity during the Second World War (WWII), the United States (US), United Kingdom, France, Japan and other countries invested in increasing production and research capacity, laying the foundations of what would become the post-WWII pharmaceutical sector. For decades this sector was characterised by large vertically-integrated firms based in a handful of the most industrialised countries, often working alongside government and university researchers and benefiting from publicly-funded science. But starting in the 1970s scientific advances opened the way for smaller firms specialising in biotechnology and working closely with academia (Achilladelis & Antonakis, 2001; Dutfield, 2020; Malerba & Orsenigo, 2015).

More recently, the centre of gravity of innovative activity has shifted away from the largest firms towards small/medium enterprises (SMEs) and the academic labs from which many of them originate(Geilinger & Leo, 2019; Lincker et al., 2014; Navarro et al., 2019; Pammolli et al., 2020). A 2019 study estimated that 33% of the forecast sales for the 12 biggest pharmaceutical companies derived from acquisitions rather than in-house inventions (Deloitte Centre for Health Solutions, 2019). While large multinational firms continue to dominate late-stage R&D, production, pricing and supply, most new medicines obtaining regulatory approval originated from SMEs, which are also increasingly obtaining regulatory approval themselves (Geilinger & Leo, 2019). Currently,

Furthermore, a growing number of countries, including both industrialised and developing, are investing in strengthening their pharmaceutical R&D capacity (Vieira et al., 2023). The contemporary PIS has evolved into a complex global mix of universities, small/medium/multinational firms, contract service providers, government and philanthropic funders, regulators and patient and other civil society groups in high, middle and low-income countries (Henderson et al., 2006; McKelvey & Orsenigo, 2001; Vieira et al., 2023).

What explains the evolution of this system? Three major factors emerge from the literature: rules (e.g. laws and regulations), financial resources, and scientific or technological change. These factors can act as exogenous or endogenous ‘shocks’ driving change in the system (McKelvey & Orsenigo, 2001).

For example, significant and lasting changes in the sector have been spurred by changes in national rules such as the more stringent regulatory standards on product safety and efficacy introduced by the 1962 Kefauver-Harrison amendments in the US (Greene & Podolsky, 2012), or the US 1982 Bayh-Dole Act that sought to facilitate collaboration between academic researchers and industry (Tseng & Raudensky, 2014). In addition to national laws and regulations, scholars have explained how the globalisation of rules – particularly IP but also regulatory standards – has expanded markets for established pharmaceutical firms and shaped national innovation pathways (Chang, 2002; Chaudhuri, 2005; Hoen, 2009; Kamiike, 2020; Lindström-Gommers & Mullin, 2019; Sell, 2003). The adoption of national rules modelled off the US Bayh-Dole Act in many countries is another important example of how rules have changed innovation systems (Gores & Link, 2021; Hemel & Larrimore Ouellette, 2017; So et al., 2008).

Financial resources are another important driver of system change. On the demand side, the creation of welfare states providing healthcare to populations in post-WWII Europe expanded markets for medicines, giving firms incentives to invest in production and innovation (McKelvey & Orsenigo, 2001). Financial success with a product could solidify the position of major firms, allowing them to invest in maintaining a dominant position in a particular therapeutic area (Achilladelis & Antonakis, 2001). On the supply side, increased public investment in scientific research at universities and other research institutions laid the foundations for innovation by expanding basic understanding of biology and disease, as well as directly funding some companies (Cleary et al., 2020; Galkina Cleary et al., 2018; McKelvey & Orsenigo, 2001).

Scientific and technological advancement has been recognised as the third main category of drivers of change. Beginning in the 1970s, new understandings of molecular biology gave rise to the ‘biotechnology revolution’, with the search for medicines expanding beyond small-molecule chemicals to more complex biologic products such as proteins. One consequence was the need for closer collaborations between cutting edge academic researchers and the pharmaceutical industry, and the emergence of small firms as ‘spin-offs’ from academic institutions (Henderson et al., 2006). More broadly, the increasingly important role of academic researchers, the rise of new biotech firms, and the emergence of venture capital willing to invest in these firms, expanded both the number of actors in the system and the complex networks of relationships between them (Dutfield, 2020; McKelvey & Orsenigo, 2001). New therapies were being developed as a result of networks rather than from single vertically-integrated firms alone, an important shift in the system that continues to this day (Lincker et al., 2014; Moon et al., 2022; Navarro et al., 2019).

Scholars highlight how these three categories of drivers – rules, financial resources and scientific technological advances – interact and co-evolve (Dutfield, 2020; Henderson et al., 2006; Malerba & Orsenigo, 2015). For example, changes in *rules* regulating prices could expand or reduce the *financial resources* available to firms. Changes in *financial resources* such as public investment drove the *scientific and technological advances* that gave birth to new biotechnology firms. And those *scientific advances* shaped the development of new *rules*, such as the Bayh-Dole Act, which then incentivized academic-industry collaborations.

In addition to scholarship on the PIS as a whole, a rich literature also examines alternative approaches to innovation in primarily four areas: pandemic preparedness (Matheny et al., 2008; Sunyoto, 2020), neglected diseases (Bottazzi & Brown, 2008; Moran, 2005; Munoz et al., 2015; Vieira et al., 2022), rare diseases (Alonso Ruiz et al., 2023; Daniel et al., 2016; Douglas et al., 2022; Heemstra et al., 2009) and antibiotics (Jaczynska et al., 2015; Outterson, 2014; O’Neill, 2016). This research often diagnoses the problems each niche emerged to address and proposes solutions to them. Such problems are often characterised as market failures (Abi Younes et al., 2020). However, critics have argued that the broader concept of ‘system failure’ is more appropriate than ‘market failure’, which could be understood to suggest that the problem would be solved if the market can simply be ‘fixed’ (by, for example, creating larger market incentives or changing market rules) (Geiger & Gross, 2018; Mazzucato, 2016).

The literature also describes and analyzes the origins of each niche and initiatives that populate them. But scholarship usually focuses on a single niche or specific initiatives, rather than explaining more generally how niches emerge in the broader PIS or comparatively analyzing niches with each other.

This article seeks to contribute to the literature in several ways. First, the literature on medicines pricing has largely focused on factors influencing pricing and policy interventions *after* a product has been developed. (We do not review this literature here due to space constraints, but flag here a relatively recent systematic review [Tordrup et al., 2020].) We complement the pricing literature by focusing on how changes to the pharmaceutical innovation model itself could deliver fairer prices. Second, the literature often characterises the PIS as a single (albeit complex) one that may exhibit variation across countries or evolution over time, but has not yet adequately accounted for unique sub-systems that emerge within it. Our paper offers a novel conceptual framework of the PIS as a forest that incorporates the emergence of specialised niches created to address weaknesses in the mainstream system. We build on Geels’ conceptualisation of niches in socio-technical systems as protected spaces where radically-different technologies can be developed (Geels, 2004), expanding the concept to spaces that do not just produce different technologies but where the processes of technological innovation themselves are different; where, as Geels’ described it, ‘it is possible to deviate from the rules in the existing regimes’ (2004, p. 912). Third, this paper then offers a theory for how and why such sub-systems emerge and evolve, adding to the literature’s focus on rules, financial resources and science-related drivers a consideration of societal norms and the creation of new actors. We integrate insights from the study of complex adaptive systems (CAS), which provide further theoretical foundation for our arguments regarding the diverse and non-deterministic drivers of change in the system (Borghi et al., 2022).

## 2. Methods

The development of the conceptual framework and theory of niche emergence and evolution were largely derived inductively, and are the result of several strands of research in a five-year research project on pharmaceutical innovation models. We began by conducting literature reviews on the mainstream PIS, its history, actors, and rules that shaped the system.^2^ Having observed that the system exhibited traits of a CAS, we constructed a novel conceptual framework (Section 4.a) using concepts and insights from CAS. We then iteratively revised this framework as the project’s empirical research progressed, and tested its suitability across multiple niches.

We also mapped initiatives that could potentially meet the project definition of a ‘new or alternative business model’: an initiative that funds, implements or facilitates R&D in a manner that differs significantly from the mainstream innovation model, and with the aim of better meeting global public interest objectives such as fair pricing or missing innovation. We defined the mainstream innovation model as a market-driven one in which a commercial profit-maximising firm conducts at least the later stages of R&D (e.g. preclinical to clinical trials) and brings a product to market. Competition between companies and market incentives influence which diseases or technologies the firm prioritises; how it manages knowledge such as data and intellectual property; and its strategies for obtaining regulatory approval, production, marketing, distribution and pricing. Historically, these firms have been based in the most advanced industrialised countries, though the same business model is now emulated by firms in middle-income countries such as China or India.

AIMs differ from the mainstream one along one or more of the following dimensions: an organisation’s mission, priority-setting process, organisational form, approach to financing, role in different phases of the R&D process, approach to knowledge management (including but not limited to intellectual property), approach to regulatory standards, manufacturing strategy, distribution strategy, and pricing of final products. We found a total of about 140 such initiatives (database available online^3^). As we gathered this data, we observed that a critical mass of the initiatives were clustered around similar disease or technology areas, which we began to characterise as *niches* within the broader ecosystem.

We then focused on each of the four niches, conducting literature reviews to understand how they had emerged, and the norms and rules, resources^4^ and actors within them. In addition to background data from publicly available sources on each of the 140 initiatives in the database, we conducted 53 interviews representing 47 organisations from 2020 to 2023. We also held four online workshops in 2023 with interviewees and other experts for each of the niches to solicit feedback on our initial findings, with a total of 52 attendees. This paper offers a high-level comparative overview of the niches; detailed analyses of each niche are presented elsewhere.^5^

Finally, we identified from the literature an initial list of drivers of change in the PIS, which we then supplemented with additional drivers found in each niche. We compared the four niches to identify how different factors shaped niche emergence or evolution.

## 3. Results

We first describe the conceptual framework, then present how each of the four niches emerged and evolved.

### 3.1. Conceptual framework: pharmaceutical innovation as a complex adaptive system (forest)

A central goal of our research was to deepen understanding of how the PIS may change to better address global public health needs, including by delivering fair prices and needs-based innovation by design. Doing so required developing a model of the system itself, which we defined as comprised of three elements: *resources, actors* and the *norms and rules* that govern their interaction, building on previous work on socio-technical systems in general (Geels, 2004) and the global health system in particular (Szlezak et al., 2010). We conceived of the R&D system as a global one, with national innovation systems embedded within it. The system is also complex, encompassing thousands of actors, billions of dollars, and hundreds of rules operating simultaneously at organisational, sub-national, national and global levels. Actors within this system behave strategically, adapting to changes in their environment (e.g. decisions of competitors, changes in regulations, advances in science), thus making the system not only complex but also adaptive. Therefore, we applied insights from the study of complex adaptive systems (CAS) to facilitate analysis.

Combining the concepts of a CAS with a PIS yields a Complex Adaptive Pharmaceutical Innovation System (CAPIS). We define the CAPIS as a group of actors that interact in a sustained way through time to produce certain outcomes (e.g. the successful development of a new medicine, the extent of its population health impact). Norms and rules operating from local to global levels shape how actors behave, and actors’ strategic adaptation makes the system’s evolution non-linear and difficult to predict. The system can be resistant to change and exhibit lock-in (due to self-regulating negative feedback loops), or it can experience tipping points (due to positive feedback loops) that change the system in significant ways. Complexity implies there can be multiple drivers of system stasis or change (Borghi et al., 2022; Hill, 2011; Holland, 1992).

Comparing the CAPIS to a forest can illuminate how it functions (see summary in Table 1). We characterise various R&D actors in the system as different organisms (e.g. types of mushrooms, trees or ferns) falling into three categories:

1. *Actors (i.e.* organisations of individuals):
  -  (a) *Implementing organisations (e.g. mushrooms)*: those who conduct R&D, including academic labs, hospitals, academic spinoffs and other SMEs including biotechnology firms, multinational firms, non-profit research organisations including some product development partnerships (PDPs), public benefit corporations (PBCs), contract research organisations, contract manufacturers
  -  (b) *Financing organisations (e.g. trees)*: those providing push or pull funding for R&D, including venture capital, private equity investors, small and large investors in equity markets, philanthropic foundations, governments, public or private insurers, and consumers of health technologies
  -  (c) *Governing organisations (e.g. ferns):* a broad category including organisations that influence R&D, without conducting it themselves or paying for it. It includes actors who make, apply or interpret relevant laws such as regulators (i.e. national, regional, global regulatory agencies [e.g. European Medicines Agency, African Medicines Agency, the World Health Organization], legislators, patent offices, and the judicial system). It also includes facilitators who seek to advance or otherwise improve the R&D process, including patient organisations, technical assistance providers, ‘matchmakers’, and ‘system integrators’ such as PDPs or government agencies. Finally, we also include in this group actors who influence innovation through other channels, such as journalists, experts, advocates, or civil society organisations. These actors may shape priority setting, policy making, resource mobilisation and allocation, and the practices adopted by other actors.

**Table 1.** Summary: the complex adaptive pharmaceutical innovation system (CAPIS) as a forest.

Forest	CAPIS
**Organisms**: e.g. mushrooms, trees, ferns	**Actors**: Implementing, Financing, Governing
**Resources**: Sunlight; water; soil; air	**Resources (types of capital)**: knowledge (intellectual capital); financing (financial capital); manufacturing (physical capital); relationships (social capital)
**Laws of nature:** gravity, inertia, entropy	**Rules and norms** shaping resource flows and actor interactions in the system: Laws and regulations: intellectual property rules, regulatory rules, tax, reimbursement, formularies Societal norms: social purpose, who should benefit, priority-setting, ethical values
**Niches**: ponds, dead logs, meadows, tree canopy	**Niches**: pandemic preparedness, neglected diseases, rare diseases, antibiotics, others
**Outputs**: fruit, flowers with certain characteristics (e.g. sweet, sour, large, small)	**Outputs**: new medicines with certain characteristics (e.g. more or less safe, effective, affordable, available, adapted for context/end-user).

These categories are not mutually exclusive. A financing organisation can also be a governing organisation, such as when a government agency seeks to accelerate R&D by both funding it and providing technical support to implementers. Or, an implementer may be its own financier, which is the case of large firms that have earned profits and use them to pay for R&D. We have found the different categories are analytically useful, nevertheless, for highlighting the different roles that actors play.

In a forest, access to resources like sunlight, water, soil nutrients and air shape which organisms wither or thrive. Similarly, in the CAPIS, access to various resources is necessary to conduct R&D and influences the emergence, survival and/or success of different actors. We inductively identified four kinds of resources (or ‘capital’ [Bourdieu, 1986]) that implementers need to conduct R&D:

2. *Resources:*
  -  (a) *Knowledge (sunlight, i.e. intellectual capital).* Knowledge embodied in human resources (e.g. scientific expertise, regulatory experience, manufacturing know-how, management capacity), as well as disembodied knowledge that is (more) easily transferable (e.g. data, manuals, publications, candidate compounds, intellectual property rights).
  -  (b) *Financing (water, i.e. financial capital*)*:* Financing that pays for R&D upfront (through push funding such as grants, investments) or by promising financial returns at a later stage (through pull incentives such as expected revenues from product sales, procurement mechanisms, and additional financial rewards).
  -  (c) *Manufacturing capacity (soil, i.e. physical/material capital)*: Capital required to produce pharmaceuticals, such as raw materials, machinery, and production facilities.
  -  (d) *Relationships (air, i.e. social capital)*: Interpersonal relationships that actors use to acquire the other three resources. These may depend on reputation, experience, political influence, personal networks of leaders, trust and the longevity of relationships, among other factors.

But a forest is more than resources and the organisms that depend on them. It is also defined by the ‘laws of nature’ that govern their interaction, such as gravity, inertia or entropy. Likewise, the CAPIS is not only defined by its actors and resources, but by the formal rules and societal norms that govern their interaction:

3. *Rules & norms:*
  -  (a) *Laws and regulations:* Legal rules that usually operate at the national level and include regulatory standards and requirements (including research ethics), tax policies and intellectual property rules. They are explicitly articulated, formally binding and legally enforceable through national institutions such as courts, regulators and tax offices (Geels’ ‘regulative and formal rules’ [2004]).^6^
  -  (b) *Societal norms:* Norms are less clearly-articulated than laws and regulations, but shape innovation processes by establishing what is socially-acceptable or desirable (Geels’ ‘normative rules’ [2004]). Examples of relevant social norms include those on the public purpose of R&D, ethical values on who should benefit from it, what should be a research priority or how it should be decided, and what are reasonable prices or profits from R&D. Violations of the norms may not be enforceable in the same way as formal rules, but they impose costs such as reputational harm or social opprobrium (Finnemore & Sikkink, 1998).

Rules and norms can influence the behaviour of actors and the flow of resources, and thereby profoundly shape how the system functions. For example, market-based incentives such as IP orient the system towards more lucrative diseases, neglecting health needs that are less profitable. Rules and norms are not static, however, and are also shaped by actors and the resources they control.

**Figure 1.**
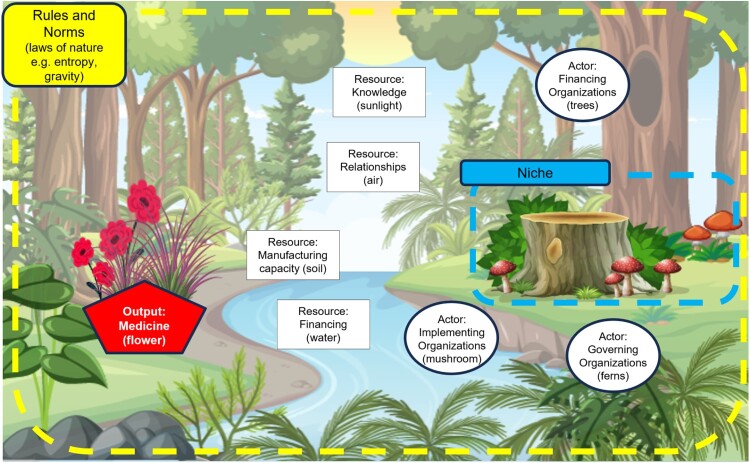
Complex adaptive pharmaceutical innovation system as a forest. Source: Bétina Zago.

Within a forest there are smaller-scale levels of organisation, such as the niche ecosystem of a dead log or a pond (Figure 1). While both make use of the same resources (sunlight, soil, air and water) each is occupied by a set of organisms interacting in specific ways. Similarly, in the CAPIS we have identified certain niches, populated by a specific mix of actors, resources and sometimes specialised norms or rules. In some cases, these involve the same actors as in the mainstream system (e.g. multinational firms), but in others new actors emerge such as PDPs or PBCs. Sometimes resources are the same as in the mainstream system (e.g. private investment for some rare disease drugs, or knowledge acquired through market transactions), while in others resources differ (e.g. public or philanthropic funding, or openly-shared knowledge).

Bringing all of these components together, we posit that just as certain conditions allow a tree to produce smaller or larger flowers that smell more or less fragrant, the interaction of these factors can result in medicines that perform better or worse at meeting public needs, for example, via the diseases they address, their affordability or suitability for use in certain contexts.

### 3.2. How do these components work together in the mainstream innovation system?

We consider the mainstream market-driven innovation system as the baseline against which to compare how alternative niches function within it. The system has evolved considerably over the past decades, but market incentives remain an enduring feature. From the 1950s–1990s, large vertically-integrated pharmaceutical firms came to dominate the system as earnings from successful products allowed certain firms to consolidate their positions within the market (Achilladelis & Antonakis, 2001). However, over the past several decades many of the larger firms have reduced in-house R&D investment, acquiring SMEs or in-licensing their technology at later – less risky but costlier – phases of R&D (Tulum & Lazonick, 2018). In addition, non-commercial actors such as universities, hospitals and other research institutions increasingly lead R&D projects targeting smaller patient populations (Pammolli et al., 2020). The R&D process now often resembles a relay race, with many different public, commercial and non-profit ‘runners’ potentially involved at different stages. For example, a scientist at a publicly-funded academic institution may discover a promising compound for a disease, then spin-off a start-up firm to advance development further. This start-up may advance the candidate a few more steps into preclinical testing before out-licensing it to another developer or being acquired by a mid-size firm. The mid-size firm may further develop the product through Phase 1 and 2 trials, then out-license it again to a large multinational, which may complete Phase 3 trials and seek regulatory approval. (Or, the SME may take the product all the way through to the market.) Manufacturing may be contracted out to another firm, and marketing and distribution rights sold to different firms in different countries. The relay may not always be a single, linear race, but rather, a single promising compound can be out-licensed to multiple runners for different diseases or country markets, and move in different directions at different paces.

While recognising these complexities and heterogeneity in the pharmaceutical innovation process, both the traditional vertically-integrated firm and the more recently-emerged relay-race approach remain primarily market-driven. Maximising profits and returns to investors shapes each decision along the long route from drug discovery to patient.

In contrast, each of the four niches we have identified is characterised by either non-market end goals (e.g. national security for pandemic preparedness, health impact for neglected diseases), or a mix of market and non-market goals (e.g. limited profit for rare diseases, sustainable profit for antibiotics). This difference in core objective of the innovation system produces alternative flows of resources, different roles for various actors, and sometimes even different laws undergirding the system, as the next section discusses.

### 3.3. Models of change: emergence of niches and evolution over time

A wide range of concerns with the mainstream CAPIS has prompted the creation of at least four niches. We provide below a brief summary of the emergence and evolution of each, identifying key shifts and drivers of change:

#### 3.3.1. Pandemic preparedness

As mentioned, the modern research-based pharmaceutical industry emerged from WWII military efforts to develop and produce medicines to protect soldiers. Subsequently, what had begun as a government-driven innovation system evolved into one dominated by the private sector, with firms investing in R&D, bringing products through development to market, and retaining control of the technologies that resulted. However, there remained a small ‘mission-driven’ niche in this market-driven CAPIS to develop health technologies for biological threats to security (Sunyoto, 2020). Previously referred to as military or biosecurity R&D, it focused on emerging infectious diseases (EID) for which no effective tools existed. In the wake of the Covid-19 emergency, the niche has increasingly been referred to as ‘pandemic preparedness and response (PPR)’, which is therefore the terminology we use here.^7^

The literature suggests the United States’ PPR R&D system is the largest and most well-developed (Sunyoto, 2020). For example, US military research developed vaccines for smallpox, yellow fever, tetanus, influenza, and viral hepatitis (Sunyoto, 2020). After the September 11th, 2001 attacks on the US, the government created the Biomedical Advanced Research & Development Agency (BARDA) with the mission of developing new products for threats like anthrax that could harm civilians as well as soldiers (Houchens & Larsen, 2017). BARDA funding was responsible for the development of 51 products from 2007 to 2020 (e.g. for influenza, anthrax, Zika, Ebola and others, see Sunyoto, 2020 for a list). Other countries with notable military investment in infectious disease research include France (Binder, 1999), the UK (Bailey, 2013; Herron & Dunbar, 2018), Germany and Russia (Shanks et al., 2016).

Prior to Covid-19, there was already significant growth in this niche: the number of active companies grew from 133 in 2008 to 303 in 2016; mostly SMEs (accounting for 86% of products in the pipeline), and based in a few countries (e.g. the US, China, UK, Canada and Switzerland) (Milne, 2019; Milne et al., 2017). By 2019, EID R&D investment had reached USD 1.44 billion, 88% of which was public funding with 57% of the total from the US government alone (Policy Cures Research, 2020). R&D investment for Covid-19 dwarfed these figures, for a total of USD 11.2 billion over 2020 and 2021, of which 73% was public (the US remained the largest public funder, but its share dropped to 39% as many other governments also invested).^8^

Because it is impossible to predict when an EID outbreak will occur or how far it will spread, commercial markets for products only materialise when a large-scale outbreak occurs, making R&D investment too risky for the private sector (Smith et al., 2003). The public sector therefore takes a leading role by funding and facilitating the innovation process. For example, policy tools in the US system include government technology transfer, grants, prizes, exclusive rights, measures to facilitate regulatory approval, and procurement for stockpiles (Gross & Sampat, 2021; Matheny et al., 2007). The European Union used large-scale advance market commitments to secure Covid-19 vaccines prior to regulatory approval, which de-risked R&D and production scale-up for companies (Alonso Ruiz et al., 2024).

The approach to PPR R&D can be summarised as follows: Public authorities identify current and future threats, set priorities for technology development, invest public funds directly in R&D conducted by public and private actors, provide incentives for private investment, and also engage directly in R&D activity. PPR R&D was built on the historical legacy of military R&D, with sustained investment from government budgets. It has traditionally focused on invention, with almost no attention to ensuring global affordability or availability of the technologies that result.

This trend shifted with the creation of the Coalition for Epidemic Preparedness Innovations (CEPI) in 2017 as a global partnership in which public, philanthropic and civil society organisations aimed to accelerate the development of vaccines for EID. The creation of CEPI was prompted by the 2014–2015 West African Ebola crisis, for which promising vaccines had been partially developed but were sitting on a shelf, unavailable for use to control the epidemic (Gouglas et al., 2019). Notably, the creation of CEPI aimed not only to accelerate vaccine development but also to make them globally accessible – that is, both available and affordable – when an outbreak occurred, reflecting the rise of what has been called elsewhere the ‘access norm’ – the norm that lifesaving health technologies should be available to everyone who needs them, propelled by the HIV/AIDS crisis at the turn of the millenium (Hein & Moon, 2016).

In the wake of COVID-19, governments mobilised resources and created new actors for PPR R&D. Notably, the European Union created in 2021 the Health Emergency Preparedness and Response Authority (HERA) and Japan in 2022 the Strategic Center of Biomedical Advanced Vaccine Research and Development for Preparedness and Response (SCARDA). Both are modelled on the US BARDA system, prompted by the wake-up call that neither Europe nor Japan had dedicated pandemic R&D programmes when Covid-19 first struck (European Commission, 2021; Normile, 2023). Developing countries are also investing in strengthening PPR R&D capacity, including in Brazil, Indonesia and Rwanda for the African continent. Several open science initiatives were also launched to make pandemic products more accessible by design, largely based in academic institutions. In 2021, governments launched negotiations towards a pandemic treaty whose scope includes pandemic-related R&D, production and access, an effort to change international rules governing the niche.^9^

A more detailed description and analysis of this niche is available elsewhere,^10^ here we provide a summary of key steps in the emergence and evolution of the niche.

The formation of this niche can be traced to a *social purpose* that began as national security and evolved over time to encompass both rapid invention for potential pandemics coupled with more equitable global access to those inventions. A series of shocks prompted the mobilisation of *new public resources* to create *new actors*, such as BARDA, CEPI or HERA, each of which was charged with making targeted public investments in R&D for pathogens of pandemic potential. The number of private firms active in this niche had grown considerably before Covid-19, and with the new public *resources* now available, *more actors* can be expected to enter – both firms and academic, non-profit and other research actors. The importance of the access *norm* is reflected in political rhetoric and the creation of Covax, a major global initiative for equitable access to Covid-19 vaccines and other tools, though delivery fell far short of the norm (Open Consultants, 2022). The increased emphasis on improving affordability and availability is also reflected in a proliferation of initiatives to boost regionally-diversified production in the wake of the Covid-19 crisis, reflected in initiatives such as the WHO mRNA technology transfer hub, African Vaccine Manufacturing Accelerator (AVMA) and Regionalized Vaccine Manufacturing Collaborative. A change in international *rules* is currently under negotiation, though it remains to be seen how far they will differ from the status quo.

#### 3.3.2. Neglected tropical diseases

As noted, the modern pharmaceutical innovation system emerged in the most advanced industrialised countries, and did not prioritise diseases predominant in developing countries unless important for protecting wealthy nations’ soldiers (e.g. malaria). The lack of inventions to address the so-called ‘neglected tropical diseases’ began to attract resources and prompt the creation of new actors in the 1960s and ‘70s. For example, the Fogarty International Center was established in 1968 at the US National Institutes of Health to conduct research on health challenges of the developing world (Desowitz, 1991). In the UN system, in 1975 the Special Programme for Research and Training in Tropical Diseases (TDR) was created (Daar et al., 2008). These initiatives advanced scientific understanding of diseases primarily affecting developing countries through research funding, projects and capacity building programmes, and successfully developed several essential technologies such as insecticide-treated bednets to prevent malaria and ivermectin to treat river blindness (onchocerciasis). However, by the 1980s it became clear these efforts were insufficient to address the spread of drug-resistant malaria and tuberculosis, and the rapidly-spreading HIV epidemic (Moon, 2009).

Health R&D rose on the international agenda throughout the 1990s due to a series of reports that re-framed health technologies, not as regular private goods that should be left to market forces, but rather as essential goods for advancing human development that therefore merited greater public involvement. The 1990 report of the Commission on Health Research for Development argued that research was ‘under-recognized and neglected’ as a tool for addressing growing global health inequities and urged greater investment in health research in developing countries (The Commission on Health Research for Development, 1990). Shortly thereafter, the 1993 World Bank report Investing in Health provided evidence that health was an essential pre-cursor (not only a desirable outcome of) economic development (Musgrove, 1993), and the follow-up 1996 report Investing in Health Research and Development, called specifically for increased investment in health technology R&D (WHO Ad Hoc Committee on Health Research Relating to Future Intervention Options, 1996). Finally, the 1999 publication of the 10/90 Report on Health Research by an expert group added an overtly normative dimension to the debates, arguing that investing only 10 percent of the world’s R&D funding on diseases primarily affecting 90 percent of the population was an unethical and unacceptable imbalance (Global Forum for Health Research, 1999).

A response to this shift in expert opinion was the creation of new actors. From the late 1990s, approximately two-dozen new organisations were created to drive R&D into neglected diseases (Moran, 2005; Moran et al., 2012; Widdus, 2001; Ziemba, 2005). They collectively came to be called public-private product development partnerships (PDPs), and reflected a broader turn in global health towards public private partnerships (Buse & Harmer, 2004; Lidén, 2013). Mainstream market-driven R&D models did not and would not generate innovative technologies for these diseases (Trouiller et al., 2002); at the same time, R&D capacity was understood to be largely concentrated within the pharmaceutical industry, and finding ways to channel that capacity for public health goals became a key objective.

While there is significant variation in how they operate, a PDP is usually a non-profit organisation that acts as a ‘system integrator’ to advance R&D by bringing together academic, government, industry and philanthropic actors to develop jointly new health technologies directed at unmet health needs (Munoz et al., 2015). PDPs are generally funded by public development assistance and philanthropic contributions. PDPs as a group have demonstrated that it is possible to develop affordably-priced medicines through AIMs, as evidenced by significant increases in funding for neglected diseases R&D, a renewed pipeline and over 50 products developed by PDPs in the last two decades for diseases such as tuberculosis, malaria, leishmaniasis, Chagas disease, sleeping sickness, cholera and Ebola, now reaching patients (see Vieira et al., 2022 for a list). Products are designed from the earliest stages of R&D to be affordable, available and well-adapted for use in resource-poor settings (Munoz et al., 2015; Vieira et al., 2022).

In 2006, also in response to the problematisation of neglected disease R&D, a new rule was created in the US that would mobilise new resources and make possible other innovation models for neglected diseases – the priority review voucher (PRV). PRVs are tradeable vouchers for expediting regulatory review of a product, and subsequently reduce the time for a medicine to enter the market (Ridley et al., 2006). By selling a PRV to a private firm, neglected disease drug developers can earn a financial reward for their R&D investment. PRVs were originally conceived for neglected disease medicines in 2006, then subsequently expanded to include rare paediatric diseases in 2012 and pandemic preparedness products in 2016 (Mezher et al., 2020). As of 2019, 31 PRVs had been granted by the US FDA of which 17 had been sold for USD 67.5-350 million– significant sums that could finance R&D in a smaller organisation for at least several years (Dicken, 2020).

A more detailed description and analysis of this niche is available elsewhere,^11^ here we provide a summary of key steps in the emergence and evolution of the niche:

Overall, the neglected disease niche operates on different norms and rules than the mainstream CAPIS. Funding is largely public and philanthropic, cooperation between R&D actors is common, and products are affordable by design. This niche began to emerge in response to changing *societal norms* in the 1960s–70s, which led to the creation of *new implementing and funding actors* and the mobilisation of *new (largely public) financing*. After a period of successes and failures, shifts in ideas and *norms* again drove the creation of *new governing actors* (the PDPs), which mobilised again *new financial and knowledge resources* for neglected disease R&D. These same *norms* also led to the creation of at least one *new rule* – the US PRV – that mobilised *new financial resources*, and may make feasible a limited-profit R&D model alongside the largely non-profit R&D model that dominates the niche; that is, *new implementing actors* are starting to enter the field.

#### 3.3.3. Rare diseases

Rare diseases comprise another niche that has evolved separately from the mainstream CAPIS. As rare diseases affect a relatively low number of potential patients, markets were considered too small to attract sufficient private R&D investment. To address this problem, US patient associations (e.g. American Muscular Dystrophy Association) and other key individuals created the National Organization for Rare Disorders, and advocated in the 1970s and ‘80s for specific legislation to address the lack of treatments for these diseases (Huyard, 2009).

As a result, the US Orphan Drug Act (ODA) was passed in 1983, the first of its kind worldwide; its key features have since been adopted in over 90 countries (Chan et al., 2020), including in the EU in 1999 (Regulation (EC) No 141/2000 on orphan medicinal products). The specialised rules in US and EU law include market monopolies (US, EU), grants for clinical trials (US, EU), tax credits (US), centralised registration (EU), and other technical assistance and benefits from regulators such as fee waivers (Sarpatwari et al., 2018; Westermark & The Committee for Orphan Medicinal Products and the European Medicines Agency Scientific Secretariat, 2011). In other words, the new rules created a range of new *resources* to support R&D, from direct push funding (grants, tax credits) to knowledge (technical assistance) to pull funding (longer monopolies, decreased fees).

From 1983 to 2022, the US FDA approved 1122 orphan designated products, 882 of which were approvals for a first Orphan Drug Designation (Fermaglich & Miller, 2023). Between 2000 and 2020, the EMA authorised 142 orphan medicines (European Medicines Agency, 2021), of which it was estimated that the regulation was directly responsible for the development of about 18–24 (European Commission, 2020). These new rules were considered so influential that the ‘niche’ has grown into a large proportion of the mainstream pharmaceutical innovation system: from 2018 to 2022, 53% of new drug approvals in the US qualified at time of registration as orphan drugs.^12^ In addition, these new rules made it more feasible for SMEs to bring products to market by making it possible to obtain regulatory approval with small numbers of participants in clinical trials (Lincker et al., 2014; Wellman-Labadie & Zhou, 2010). Other scholars have suggested that orphan drug laws changed the structure of the industry such that SMEs focus on earlier, riskier stages of R&D while large firms focus on the later, less risky stages of development, production and marketing (i.e. a relay race) (Heemstra et al., 2008; Valverde et al., 2012; Wellman-Labadie & Zhou, 2010).

Despite success in developing some new treatments, this niche still suffers from many shortcomings. To date only 5% of rare diseases have a treatment option (Horgan et al., 2020). Scholars have found uneven effects of the EU regulation, with many new products for oncology, but unmet needs for other pediatric and rare diseases (de Jongh et al., 2018). In addition, the rules have had some unintended effects, with very high prices foremost among them. A 2019 study found that R&D costs for orphan drugs were on average significantly lower than for non-orphan drugs (Jayasundara et al., 2019), but average annual prices of orphan drugs were 25 times more expensive than non-orphans in the US (AHIP, 2019). High prices for orphan drugs are justified on the grounds that sales volumes are low; however, one study found that gross profit margins for orphan drugs were over 80%, while the pharmaceutical industry average was 16% (Phillips, 2013). Another study found that orphan drugs also generate about a 10% higher return on investment than non-orphan drugs (Hughes & Poletti-Hughes, 2016). Orphan designations were found to result in ‘overcompensation’ to developers (European Commission, 2020). In addition, the possibility of applying for several orphan drug designations for one product, or of sub-dividing a disease into smaller categories in order to qualify for orphan drug designation (‘salami-slicing’) have also been flagged as misuses of these incentives because they do not result in additional innovation (de Jongh et al., 2018; Sarpatwari & Kesselheim, 2019).

The ongoing lack of innovation to address most rare diseases, coupled with deep concern about high prices and mis-applied incentives – in other words evolving norms about what is appropriate – prompted the creation of new actors that may continue to push forward the evolution of the niche (Douglas et al., 2022). We identified 15 organisations involved in implementing AIMs for rare diseases. All operate under different norms from the mainstream system, as their mission is to develop new medicines for rare diseases while keeping prices affordable. As a group, they rely not only on private investment, but also philanthropic, public or impact investors. Some partner closely with patient organisations to tap into their knowledge and networks, which can accelerate R&D through improved trial design, faster recruitment of trial participants, access to funding and advocacy for regulatory approval. In general, they take a more collaborative approach than the mainstream competitive model, and engage in open science practices such as data- or knowledge-sharing to move the science forward faster. These implementing actors take different organisational forms, including not only traditional for-profit companies, but also non-profit entities, limited-profit (e.g. PBCs), and academic research centres. One example is the publicly-financed academic Hospital Clinic Barcelona, which succeeded in developing a hospital-based CAR-T therapy to treat rare forms of leukemia at a price two-thirds lower than comparable treatments from the pharmaceutical industry.^13^

A more detailed description and analysis of these alternative rare disease R&D initiatives is available elsewhere.^14^ Here we provide a summary to highlight how the niche may continue to evolve, as these initiatives are relatively new and small-scale, and it is too early to assess their impact: Overall, the rare disease niche operates on different norms, rules and resources flows than the mainstream CAPIS. The sequence of events that formed this niche can be summarised as follows: political mobilisation by patients led to the 1983 US ODA and similar new *rules* in other countries, which mobilised new financial and knowledge *resources* for R&D; these *resources* made possible the emergence of some new *actors* (i.e. SMEs) and new products for some rare diseases, albeit at very high prices. Dissatisfaction with the state of rare disease innovation was a reflection of strengthening *norms* regarding the importance of affordability and more equitable access to medicines, and this motivated entrepreneurial R&D practitioners to create new *actors.* These actors have mobilised new financial and knowledge *resources* to develop rare disease drugs that are affordable by design (Douglas et al., 2022).

#### 3.3.4. Antibiotics: an emerging niche

Microbes can evolve to become resistant to antimicrobial agents, rendering many medicines less effective over time – referred to as antimicrobial resistance (AMR). While the problem affects a broad range of medicines, innovation challenges have been particularly acute for antibiotics for bacterial infections. Antibiotics are critical to modern healthcare systems – essential to treat common infections and safe surgery, for example. However, over the past several decades, investors and companies in the mainstream CAPIS lost interest in developing new antibiotics; many closed or divested their relevant research units. As of 2020, only four large pharmaceutical companies were conducting antibiotics R&D (Plackett, 2020). Market incentives have proven insufficient to drive adequate R&D investment for several reasons. First, the need to limit the use of new antibiotics to reduce the risk of resistance means sales volumes will be relatively low by design. Second, the market size for antibiotics is significantly smaller than for other therapeutic areas (such as oncology or cardiovascular) owing to shorter treatment courses, competition with generic manufacturers, and pricing regulations in certain countries. Finally, scientific difficulties are considerable, reflected in no new class of antibiotics having been discovered since 1987 (Brogan & Mossialos, 2016).

Over the past two decades, advocates succeeded in raising the profile of AMR in general, and the lack of antibiotics innovation in particular, reflected in discussions at the G7, G20 and WHO’s Global Action Plan on AMR in 2015 (WHO, 2015). In 2016, a number of key events signalled the formation of a new niche: ideational change, the creation of *new actors*, and mobilisation of *new resources*, each of which we briefly discuss here.

The 2016 ‘O’Neill report’, commissioned by the UK Prime Minister and Wellcome, reflected and contributed to growing ideational consensus on what needed to be done. The report endorsed arguments from the expert community on the need for new innovation models to create a long-term, stable pipeline of antibiotic candidates without relying on sales volumes or antibiotic overconsumption (Horgan et al., 2020; Jaczynska et al., 2015; Outterson, 2014). Such a new model would need to address simultaneously three objectives that were sometimes also at odds: the invention of new antibiotics, conservation of their efficacy through limiting their use (‘stewardship’), and ensuring global access to needed antibiotics (Laxminarayan et al., 2020; O’Neill, 2016). Achieving all three would require ‘delinking’ the traditional incentives (i.e. profits from high prices and/or large volumes) from the invention process, and finding alternative ways to finance, price, reimburse and/or value antibiotics.

The same year witnessed the first major discernible changes to the antibiotics innovation system with the creation of two new actors that, taken together, performed the roles of funding, facilitating and implementing actors: the Combating Antibiotic-Resistant Bacteria Biopharmaceutical Accelerator (CARB-X) and the Global Antibiotic Research & Development Partnership (GARDP).

CARB-X was created in 2016 by the US government’s BARDA programme, previously dedicated mainly to developing new products for EID, and Wellcome. It was structured as a global non-profit organisation, with funding partners now also including the UK and German governments, and Gates Foundation. CARB-X aims to develop new antibiotics for drug-resistant bacteria by providing push funding to earlier-stage R&D; grant recipients are required to develop global access plans. Since 2016, it has invested USD 303.3 million in 81 projects worldwide and also provides business, scientific and technical support to grantees (CARB-X, 2021).

Also in 2016, GARDP was created with support from WHO and the Drugs for Neglected Diseases Initiative (DNDi) as a PDP that mobilises resources, identifies promising compounds, licenses candidate products from partners, contributes to R&D, and develops partnerships with public and private actors. It aims to deliver five new treatments by 2025 with EUR 500 million. By 2022, GARDP’s portfolio included 4 antibiotic treatments in partnership with pharmaceutical companies, with agreements to expand access to these drugs in resource-limited settings (GARDP | Global Antibiotic Research and Development Partnership, 2022).

Finally, the AMR Action Fund was created in 2020, initially proposed by industry associations, WHO, the European Investment Bank (EIB) and Wellcome, with financing largely from pharmaceutical companies, Wellcome and the EIB. The AMR Action Fund complements CARB-X’s role by financing later-stage R&D to move products towards regulatory approval. It aims to invest nearly USD 1 billion to develop 2–4 new antibiotics by the end of the decade (IFPMA, 2021).

While CARB-X and the AMR Action Fund facilitate and ‘push’ R&D forward through direct funding, the niche has also witnessed the mobilisation of new resources meant to ‘pull’ R&D forward through incentives. France and Germany are permitting higher prices for novel antibiotics; the US also offers extended market exclusivity and faster regulatory review for some antibiotics. These are relatively traditional approaches to pull more private-sector R&D investment by increasing potential revenues through higher prices, and do not address stewardship or global access objectives. An alternative approach, sometimes called the ‘subscription’ model, seeks to incorporate stewardship and affordability into the innovation model by offering a lump-sum financial reward that does not rely primarily on volumes or prices of units sold (Jaczynska et al., 2015; Renwick et al., 2016). This model also has the potential to eliminate price as a barrier to access, since the number of end-users is determined by need (Moon & Erickson, 2019). Two pilots are being implemented by Sweden and the UK. Sweden’s pilot began in 2018, and offers a guaranteed annual revenue to attract companies to register and make available their antibiotics in the country, which is a relatively small, low-volume market (Gotham et al., 2021). Launched in 2019, the UK’s health technology assessment agency calculated the added value of two novel antibiotics to arrive at a fixed payment to the two supplying companies (Pfizer and Shionogi), with payments commencing in 2022 (Leonard et al., 2023). Both are quite recent, neither is expected to provide a significant additional incentive for R&D alone, but if multiple countries and/or larger markets such as the US adopt similar models, the incentive could be significantly increased.

Notably, neither pilot makes arrangements to deliver global access. This task is currently undertaken by not-for-profit actors such as GARDP, which concluded three license agreements with private firms Entasis, Shionogi and Venatorx to co-develop and/or expand access to new antibiotics in many LMICs. The O’Neill Report proposed that a market entry reward as a pull incentive for new antibiotics be conditional on the recipient of the reward making its antibiotic globally affordable and available, but this approach has not yet been implemented (2016).

A more detailed description and analysis of the emerging antibiotics niche is available elsewhere.^15^ Here we provide a brief summary to highlight how the niche has evolved:

Out of the four niches examined, antibiotics is the most recent and inchoate. After insufficient innovation was problematised and some degree of consensus reached on how to address it (O’Neill report), new funding, facilitating and implementing *actors* were created (CARB-X, GARDP, AMR Action Fund) who mobilised and provided *new resources* to those conducting antibiotics R&D. At the same time, new *rules* have been proposed that would create additional *resources* but thus far they have only been implemented at a limited scale (UK, Sweden pilots). Until such rules are implemented at a larger scale, for example in the US or across the EU, the niche may not fully develop or stabilise. The global access objective has received some but quite limited attention in the niche to date, and arguably the access *norm* for new antibiotics remains nascent. Overall, it remains unclear whether these approaches will be able to generate new antibiotics and make them globally accessible.

## 4. Discussion

We conceptualised the CAPIS as a combination of *resources*, *actors*, *rules and norms*. While the mainstream CAPIS is driven by market *resources*, *actors*, *norms and rules,* alternative niches are characterised by different *resource* flows, populated by different kinds of *actors,* operating under different *norms and rules*:

*Resources:* A niche can emerge and evolve from changes in the creation, mobilisation, or control over the four kinds of resources: financing, knowledge, manufacturing, and/or relationships. For example, following growing attention to neglected diseases in the 1990s, philanthropic foundations and governments made available *new financial resources* to boost R&D efforts, resulting in many new products being developed. By increasing collaboration between research organisations in what is usually a competitive system, entrepreneurial actors in rare and neglected diseases have made *knowledge resources* more widely available to accelerate scientific progress. And by matching companies with technical expertise, CARB-X brokers new *relationships* to advance antibiotic R&D.

*Actors:* A niche can also emerge and evolve from the creation of new types of actors, the roles they play, or how they work with each other. For example, for neglected diseases the creation of PDPs re-configured how the public, private and non-profit sectors worked together. For rare diseases, former pharmaceutical industry professionals created PBCs that allow them to price their medicines at more affordable levels than profit-maximising C corporations. Alternatively, existing organisations such as academic labs or SMEs can expand their roles by moving downstream and taking a product all the way through regulatory approval, rather than concentrating only on earlier-stage R&D, as we have witnessed in pandemic preparedness and rare diseases. Finally, actors can form new configurations, such as open-source international networks of drug discovery efforts facilitated by a coordinating (‘system integrator’) organisation, as seen for pandemic preparedness or neglected diseases.

*Norms and rules:* A change in norms or rules can create a new niche, or solidify one that has already begun to emerge. For example, the past two decades have witnessed the emergence of an access norm that essential medicines should be developed and affordable to people in all countries, reflected in part in expanded policy space to interpret IP rules flexibly (Moon & ‘t Hoen, 2020). Though not articulated in binding law, this norm has driven the creation of the neglected disease niche, influenced how the pandemic preparedness niche is evolving, and is shaping at least to a small extent the rare disease and antibiotics niches. However, such soft norms are not as reliable or stabilising as changes in binding national or international law (Hein & Moon, 2016). In contrast, orphan drug legislation not only created a new niche for rare diseases, over 40 years it has enabled the expansion of this niche to generate approximately half of new products from the mainstream system in recent years. It has also supported the rise of SMEs as competitors to large multinational firms, alongside other rule changes such as the Bayh-Dole Act, with important implications for both the mainstream CAPIS and niches within it. Also, flexibilities in regulatory rules, such as an exemption in EU law that allows hospitals to prepare and use medicines under certain conditions without EU-level regulatory approval, has allowed hospitals to engage in the development of advanced therapies for rare diseases (Joseph, 2023). Finally, changes to regulatory rules, such as options for emergency use listings, adapted regulatory pathways, or priority review vouchers have all solidified the pandemic preparedness niche by clearing the path through to regulatory approval.^16^

Because formal rules usually face high thresholds for change (e.g. a majority of legislators must approve of a new law), they contribute to negative feedback loops that generate system lock-in (Douglas et al., 2022). Other kinds of feedback loops also perpetuate the status quo: for example, high prices of rare oncology drugs generate lucrative profits, providing financial resources to firms, whose political power is then enhanced to oppose any change to orphan drug incentives. Such profits attract additional private investment into rare cancers, generating additional products sold at high prices, and the cycle continues. It becomes very difficult to change the orphan drug system. A key question is then how the system can be jolted out of lock-in, and whether positive feedback loops that push the system out of stasis can be created.

We consider each of the three system components as potential ‘levers’ that can be pulled in an effort to counteract lock-in and change parts of the CAPIS. These levers are interconnected and can create positive feedback loops. For example, new *resources* in the form of financing can enable new types of partnership configurations among *actors* to emerge. Changes in regulatory *rules* such as the PRV can create new *resources* that change what *actors* seek to do. Indeed, as shown above in each niche, pulling on one lever laid the groundwork for pulling on another, as summarised in Table 2.

**Table 2.** Niche emergence, formation and solidification: sequence of changes.

Niche	Sequence of change
Pandemic preparedness	Problematisation → new resources → new actors → external shocks → new norms → new actors → new resources → new international rules (under negotiation)
Neglected diseases of poverty	Problematisation → new resources → new actors → new norms → new actors → new resources → new rules → new actors
Rare (orphan) diseases	Problematisation → new rules → new resources → (some) new actors → new norms → new actors → new resources.
Antibiotics	Problematisation → new actors → new resources → new rules (partial, and in process) → new resources

The simplified pathways of niche evolution summarised in Table 2 highlight that problematisation was an important first step across the board, consistent with the literature on the critical role of agenda-setting in effecting policy change (Kingdon, 1995; Sabatier & Jenkins-Smith, 1993). Problematisation can occur through exogenous shocks, such as the 2001 US anthrax scare or Covid-19 crisis for the pandemic preparedness niche. Or, it can come from endogenous factors, such as the efforts of advocates, experts and political leaders, as was the case for neglected diseases, rare diseases and antibiotics.

Once an issue becomes problematised and rises on the public agenda, however, the sequence of steps by which a niche emerges or evolves is neither uniform nor pre-determined. Rather, in some cases new resources seem to have come first (pandemic preparedness, neglected diseases); in others, new rules (rare diseases), and in others new actors (antibiotics). The subsequent evolution of each niche suggests there is a constant interaction between resources, actors and rules, but not in a fixed pattern or along a single pathway. This finding is consistent with our characterisation of the pharmaceutical innovation system as a complex adaptive one, in which changes in one part of the system may produce changes in others due to complex interconnections and feedback loops between them. While we have portrayed each niche’s evolution as linear in Table 2 for the sake of illustration, a more in-depth examination of each niche may reveal that such processes are not linear, but rather interactions take place simultaneously between the three levers. For example, in the pandemic preparedness niche, the access norm was evolving in parallel with the creation of CEPI as a new actor. Similarly in the antibiotics niche, new rules in Sweden and the UK were developed prior to new resources mobilised by the pharmaceutical industry through the creation of the AMR Action Fund. An important implication is that those with the power to pull on any particular lever – resources, actors, or norms and rules – may stimulate change in the CAPIS. There is not only one pathway or one set of actors with the power to change the system.

Looking across niches offers valuable lessons in how innovation with affordability can be achieved. Among the four niches, it is in neglected diseases that considerations of affordable pricing have been integrated most systematically into the R&D process, as these products are intended for use almost exclusively in lower-income countries. The rare disease, PPR and antibiotics niches also each offer examples of achieving affordable pricing by design, but the record is more mixed and it is relatively early to draw conclusions. That said, the ways in which affordable pricing was achieved for neglected diseases is applicable to the other niches, and beyond. We found three main strategies. First, public and philanthropic funders frequently require grantee R&D implementers to achieve affordable pricing for target populations as a condition of financing, through measures such as transparency of pricing and auditing production costs (Liu & Moon, 2024). Second, from the start of the R&D process, implementers consider potential end-product price as an important criteria for selecting candidate products for further development. Developers understand that a high-cost end product is unlikely to make a health impact, and would run counter to their organisational missions, and so candidate products with little potential to be made affordable do not advance. Third, once a product has been developed, R&D implementers adopt a wide range of strategies to ensure price is not a barrier to uptake (e.g. licensing IP to multiple producers for larger volume products, cost-plus or non-profit pricing and/or tiered pricing). All three of these strategies could be applied to the other niches – and beyond – if R&D organisations commit to affordability as part of their mission, and if public and philanthropic funders are willing to provide sufficient financing with affordability requirements to make such pricing possible.

Limitations of this research include that most of the initiatives in our database were based in high-income Western countries, which is also the focus of most of the English-language literature. Our findings may not be as relevant for emerging economies such as China or India, which have recently increased their investment in pharmaceutical R&D. In addition, this research was undertaken from 2019 to 2024, but each niche continues to evolve. Findings may be out of date, particularly for the antibiotics niche, which was in flux at the time of research. Drivers of change across all niches will continue to be relevant, however. Finally, we focused on four niches but also identified innovation initiatives outside these, for example, for mental illness or microbiome therapies. Alternative pharmaceutical innovation models in these other areas, though relatively few in number, may hold important insights worth seeking in future research.

## 5. Conclusions

It is possible to change the pharmaceutical innovation system to better meet societal needs, including to deliver fair medicines prices by design. In light of the enormous size and scope of the system, the considerable political power of the pharmaceutical industry and highly-regulated nature of R&D, and therefore a tendency towards system lock-in, this conclusion is not a given (Kapczynski, 2023). Nevertheless, what we have observed is not wholesale changes to the mainstream CAPIS, but rather, the carving out of niches within it. These niches have emerged in response to the problematisation of specific shortcomings reflecting changing societal norms about the public purpose of pharmaceutical innovation.

We observed the effects of three levers for changing the system: the mobilisation of new resources, changing the roles of or creating new actors, and shifting societal norms and rules. In other words, in addition to the literature’s emphasis on financial resources, rules and scientific/technological drivers of change in the CAPIS, we found that societal norms and new actors also played significant roles.

There is no monopoly on the power to pull one of these three levers. Governments or philanthropic organisations can provide new financial resources while research organisations can offer new knowledge resources; entrepreneurial leaders can create new organisations; civil society groups and moral leaders can influence norms; and all actors can organise politically to influence laws. As Geels’ argued in his study of socio-technical systems, they are prone to path dependence and lock-in, but ‘niches are crucial for system innovations, because they provide the seeds for change’ (2004, p. 913). Similarly, in the CAPIS, larger scale change may be possible through the expansion of niches as we have seen for rare diseases, and/or purposeful action to effect larger-scale changes to norms, rules, resources and actors.

Improving our understanding of how change has already been effected within the CAPIS offers insights for how to further reform the system. In addition, this conceptual framework and theory of niche emergence may also be applicable to other sectoral innovation systems, such as for climate, digital or agricultural technologies, where better serving the global public interest remains a pressing concern.

## Data Availability

The datasets generated and/or analysed during the current study are available on the project website at https://www.knowledgeportalia.org/.

## Footnotes

1 Authors’ calculations using data from: World Health Organization. Global Health Expenditure Database. https://apps.who.int/nha/database.

2 All literature reviews are publicly available under the Research Syntheses section of the project website at https://www.knowledgeportalia.org.

3 The mapping combined data from a 2016 report on alternative innovation models (Kiddell Monroe et al., 2016), previous knowledge of the research team, snowballing, and literature reviews. The database of alternative R&D initiatives is available here, including a more detailed explanation of the methodology for constructing the database: https://www.knowledgeportalia.org/database-of-alternative-r-d-initiatives.

4 See Large et al. (in prep); Slovenski, Liu et al. (in prep); Slovenski, Alonso Ruiz et al. (in prep); and Vieira et al. (in prep).

5 Ibid.

6 We exclude organizational policies such as e.g. research funder policies on patenting or data-sharing, which we consider to fall under ‘actors’.

7 See Slovenski, Liu et al. (in prep).

8 Authors' calculations based on data from: https://gfinderdata.impactglobalhealth.org/pages/data-visualisations/allEmergingInfectiousDiseases.

9 See Slovenski, Liu et al. (in prep).

10 Ibid.

11 See Vieira et al. (in prep).

12 Authors’ calculations: 132 of the 247 total drugs approved by the US FDA from 2018–2022 were designated as orphans. Data from https://www.fda.gov/drugs/development-approval-process-drugs/new-drugs-fda-cders-new-molecular-entities-and-new-therapeutic-biological-products.

13 See Alonso Ruiz (in prep).

14 See Large et al. (in prep).

15 See Slovenski, Alonso Ruiz et al. (in prep).

16 See Slovenski, Liu et al. (in prep).

## Abbreviations

**Abbreviations:** AIMs: alternative innovation models; AMR: anti-microbial resistance; BARDA: United States Biomedical Advanced Research & Development Agency; CAPIS: complex adaptive pharmaceutical innovation system; CARB-X: Combating Antibiotic-Resistant Bacteria Biopharmaceutical Accelerator; CAS: complex adaptive system; CEPI: Coalition for Epidemic Preparedness Innovations; DNDi: Drugs for Neglected Disease initiative; EIB: European Investment Bank; EID: emerging infectious disease; EU: European Union; EUR: Euro; FDA: US Food and Drug Administration; G20: Group of 20; G7: Group of 7; GARDP: Global Antibiotic Research & Development Partnership; HCV: Hepatitis C virus; HERA: European Union Health Emergency Preparedness and Response Authority; IP: Intellectual property; ODA: United States Orphan Drug Act; PBC: Public benefit corporation; PDP: public-private product development partnership; PIS: Pharmaceutical innovation system; PPR: pandemic preparedness and response; PRV: Priority Review Voucher; R&D: research and development; SCARDA: Japan Strategic Center of Biomedical Advanced Vaccine Research and Development for Preparedness and Response; SME: small/medium enterprise; TDR: UNDP-UNICEF-World Bank-WHO Special Programme for Research and Training in Tropical Diseases; UK: United Kingdom; US: United States of America; WHO: World Health Organization; WWII: Second World War.

## Innovation system change to deliver affordable medicines

A conceptual framework of pharmaceutical innovation as a complex adaptive system (forest) and theory of change.
